# A Completed History

**Published:** 1868-09-15

**Authors:** A. A. Dunn

**Affiliations:** Chicago


### A COMPLETED HISTORY.

CHICAGO, *August* 29*th*, 1868.

*To the Editor of the* CHICAGO MEDICAL JOURNAL:

WILL you be so good, if possible, as to give room to the subjoined?

CASE OF ALBUMINURIA TREATED WITH NITRITE OF UREA.

*History.*—Dr. W—— desired me to visit Mrs. ——, in consultation with him, on the 2nd June ult., whose history was as follows:

Mrs. H——; aged 29; carpenter's wife; tall, nervous and cachectic; mother of four children, youngest 18 months old. She states that fifteen weeks ago she was seized with severe pain in the lower part of the back, and became very ill, when she called a physician, who told her she was suffering from an attack of intermittent fever; and subsequently, that the more persisting pain in the back was the result of rheumatism. Her feet now began to swell, which extended to her legs, thighs and body. Her breathing became embarrassed, compelling her to be supported upright in bed. She became so weak that she required to be lifted from side to side in bed. About four weeks after the onset of her illness, an abscess broke over the pained part in the lower region of her back, and discharged a large quantity of matter. It has continued alternately to heal, re-open and discharge ever since. The swelling in her chest has diminished of late, but her thighs, legs and feet continued the same.

A short time ago her medical attendant brought another physician to see her in consultation. Immediately after this, her husband solicited Dr. W—— to attend her.

The foregoing "case" is notably interesting; but as the "history" given hardly covers the "case," I venture to add a little to that "history."

Early in March last, I was requested to see the subject of the "case" whose "history," as written, I have quoted. She complained of febrile action during part of the day, with pain in back and limbs, and thirst, the balance of the day being passed in comparative comfort. The tongue being a little coated, I did not hesitate to pronounce and prescribe for an intermittent. Within two days thereafter, I was notified that she was suffering from pain in right arm. Whether right or wrong in the first diagnosis, upon seeing her now, an onslaught of rheumatism was unhesitatingly pronounced. Perhaps, however, the case was one of "Nephritis." For three days, remedies availed but little, although *Morphine* obtained insensibility to a considerable extent, much to the delight of the patient, as neither head nor stomach suffered from sequences. About that time, however, the inflammation abandoned the arm almost absolutely, but not the patient, as the *right hip* was forthwith assailed with terrible vehemence. (Does this bilateral feature belong to nephritis?) Now followed a condition I never saw with any other patient. She absolutely refused to be lifted for any purpose whatever. The left limb being movable, though all motion of lower limbs was painful, cloths were adjusted, as well as those caring for her could adjust them, to absorb the urine which was voided at intervals, and for a time, painlessly. Dejection was attended to in the same manner. Two days after the hip was assailed, micturition was painfully performed, the pain being upward to the region behind the pubes. During the second day after, the bladder became painful; it ceased to contract upon its contents, and I was obliged to use the catheter and repeat the operation several times during the next three days, when, though painful, the bladder resumed its propulsive functions. The urine was considerable in quantity, strongly acid, without blood and without albumen. The patient resisted the earnest importunities of the husband to remove her to another bed; but the odor becoming intolerable, I ordered her removed whether she consented or not. It was then I discovered the bed was saturated with urine where the nates had rested. I entertained serious apprehensions that the skin had become poisoned by the absorption of urine, and blamed myself that she had not sooner been forced from her bed. My fears were well based. Several obstinate ulcers formed upon the nates and the region just above. Now it was that she began to make outcries about her *back* as well as her *hips*, and as the latter became less sensitive during the progress of the "case," her *back* alone seemed to be the centre of distress; and it is to be noted that, whenever requested to point out the exact location of the pain, she invariably referred to the inflamed nates and region just above. The ulcers healed under the bountiful application of *Glycerine* continued for a fortnight, and followed, toward the last of the healing process, by an unguent. To relieve the buttocks, which had become exceedingly tender from pressure, as she could lie on neither side, I improvised a ring made of cotton batting, to place them in, the region of the kidneys receiving an unwonted pressure. This secured comparative comfort, though I anticipated evil from the circular compression. After the ulcers healed, there was tenderness, swelling and hardness of the region inflamed. During this time the thigh had become swollen, and the swelling extended to the foot in the course of a few days. Bloody urine was now observed for the first time, but disappeared without special interference, to recur after the dropsy became general, and and again subside without special treatment. Urine was still somewhat acid, and now contained albumen, which increased steadily for some time, as did the alkalinity. The left foot now showed effusion, and the abdomen was distended. This did not become enormously distended as did the lower limbs. The face showed some puffiness, but not very considerable. The chest was also invaded, but gave no evidence of it by shortness of breathing till two months after the illness commenced. The hands were the last to become dropsical, and were least so of any part during my attendance. Nearly six weeks after she was confined to her bed, a large abscess ruptured just over the lower lumbar vertebræ. I confess to not having looked at the inflamed region for several days prior to the rupture, a piece of neglect not to be copied. After several days the discharge ceased, but the abscess re-formed within a fortnight, and I opened it, putting a tent in the orifice. The abscess soon ceased to discharge, but re-formed, and I opened it again just before I abandoned the case. A huge poultice was the dressing.

As might be supposed, the pulse ranged above a hundred during a whole month, and but little below that while under my care, till toward the last, and then under the influence of *Veratrum*. In the earlier stages of the ailment the tongue became .somewhat coated, and was redder than comported with safety. Before the abscess ruptured, her stomach became quite irritable, and for a few days I suspended all ingesta save aliment, and but little of that was taken. The dropsy then gained some headway. Soon, however, the irritation subsided, and the appetite became voracious. I indulged the demands of the palate and she overdid the thing, the stomach becoming intolerant of every thing. A few days of rest sufficed to restore the tone of the stomach; she ate with avidity and bore well the diuretics and cathartics now much needed to remove the effusion of the chest. They acted thoroughly, and the relief obtained was marked and permanent in the face, hands, chest and abdomen, though not complete when I abandoned the case. As the effusion diminished, a marked diminution of albumen was noticeable, and the urine slightly reddened litmus paper, but the acid reaction did not become decided while the case was mine.

I have neglected to say she had two attacks of what may be termed pleurodynia, after the chest was invaded by the effusion, the interval being several days, and *Morphine* was administered to relieve the pain, which it did effectually. She had but little headache throughout, but was much inclined to sleep after albuminuria was established; sometimes, 'tis true, under the influence of *Morphine*, but also, either from the toxic effect of urea, or from effusion within the cranium, or both.

Her strength was at no time as much diminished as might be supposed. She did some sewing and knitted several pairs of stockings during her illness, and I once found her at the table preparing vegetables for cooking.

When the arm was assailed, I put her upon the *Permanganate of Potassa* with negative results, and suspended it when the hips became involved, endeavoring then to saturate the system with *Citric acid*, lemons being allowed *ad libitum*. They finally disagreed with her. Indeed, the red tongue was attributed to their agency. *Bicarbonate of soda* followed with negative results, unless it caused or increased the alkalinity of urine. From the commencement of the ulcerative process upon the nates, I kept her upon *Iron*, either the muriated tincture, or the phosphorated elixir, except when the stomach would tolerate nothing. Wine was used freely after debility ensued, and raw egg added to it three times a day till the egg became repugnant to her. *Quinine* was given liberally. Of that article she had a horror, as she was taking it when her arm was attacked, and she believed the *Quinine* produced the rheumatism. Her aliment I endeavored to have of the most nutritious kind. To relieve the dropsy, the changes were rung upon the diuretics, and to some extent upon the cathartics. Hydragogues, when breathing became difficult, were resorted to reluctantly, as I dreaded their effect upon the stomach; but the dyspnœa had to be relieved, so I took the chances, and won. It may be stated that peristaltic action of the bowels, during the most of the illness, was maintained with little encouraging success. The promptness with which the kidneys responded to the action of diuretics, without calling forth expressions of uneasiness from those organs, barred the presumption of the existence of active nephritis. Indeed, two weeks before I abandoned the case, I informed the husband of the probable recovery of his wife, provided granular degeneration of the kidney was not established. Of that, I told him time must determine, but I did not believe it existed. When I did abandon the case, the patient was vastly improved, save as to the abscess, and as I was firmly convinced it was connected with neither bone nor kidney, that it would finally and speedily close, I verily believed.

The prolonged illness of the patient abated no tittle of her amiability, and, having taken offence several times, finally, and unexpectedly to him, I told the husband he must get another physician, as I should not call again. This course, I was aware, left me open to just such a "history" as I have quoted, but I took it deliberately, and am not disappointed over the "history of the case."

It is probable that nothing I did for the rheumatism itself was of any avail, nor the exhibition of opiates, which did attend sensibility. There may be some doubt as to the cause of the abscess. Such results do occasionally follow or accompany rheumatism; but I am strongly imbued with the idea that the circular compression of the ring, and the previous poisoning of the urine, had no tittle to do with it.

I do not think "the most remarkable feature (in the case) was the entire disappearance of albumen from the urine." Albuminous urine does occur, though rarely, in cases of rheumatic fever, and recoveries ensue, complete and permanent. Scarlet fever is not unusually followed by albuminuria, the quantity of albumen being sometimes very great, and yet recoveries, also complete and permanent, are not unfrequent, as every practitioner of even limited experience, knows. The whole case may be considered remarkable on account of its grave complications. The pain in the bladder was a new feature to me, in such a connection, but my researches led me to the conclusion that the muscular coat of the bladder is also subject to inflammation of rheumatic character. The invasion of that coat was as interesting to me as was the existence of albuminuria; because neither occur frequently in attacks of rheumatism, and both are to be regarded as interesting complications because of that infrequency, and because they added to the severity and prolongation of the "case." But "the most remarkable feature" connected with the case, is, in my opinion, the ridiculously absurd inaccuracy of the "historv" I have quoted, so far as the early condition of the patient is concerned. The writer seems to have reached his conclusions from the most striking feature of the case when he first saw it, to-wit: the albuminous deposit. For it is difficult to believe that the patient, or her friends, would have concealed the important facts which I have communicated, had a searching interrogation been instituted. These random "histories" go a great way toward rendering medical literature uncertain and obscure, and they ought not to be *perpetuated.*

The "case" quoted can be found on page 530 of your issue, under date of August 15th, current year.

A. A. Dunn, M.D.

